# Cancerous Conditions Accelerate the Aging of Skeletal Muscle via Mitochondrial DNA Damage

**DOI:** 10.3390/ijms25137060

**Published:** 2024-06-27

**Authors:** Yi Luo, Rina Fujiwara-Tani, Isao Kawahara, Kei Goto, Shota Nukaga, Ryoichi Nishida, Chie Nakashima, Takamitsu Sasaki, Yoshihiro Miyagawa, Ruiko Ogata, Kiyomu Fujii, Hitoshi Ohmori, Hiroki Kuniyasu

**Affiliations:** 1 Department of Molecular Pathology, Nara Medical University School of Medicine, Kashihara 634-8524, Japan; lynantong@hotmail.com (Y.L.); isao_kawahara@a011.broada.jp (I.K.); ilgfgtk@gmail.com (K.G.); shota.nukaga@gmail.com (S.N.); g.m__r1@outlook.jp (R.N.); c-nakashima@naramed-u.ac.jp (C.N.); takamitu@fc4.so-net.ne.jp (T.S.); y.miya1103@gmail.com (Y.M.); pkuma.og824@gmail.com (R.O.); toto1999-dreamtheater2006-sms@nifty.com (K.F.); brahmus73@hotmail.com (H.O.); 2 Key Laboratory of Neuroregeneration of Jiangsu and Ministry of Education, Co-Innovation Center of Neuroregeneration, Nantong University, Nantong 226001, China

**Keywords:** cancer sarcopenia, aging, mitochondria, mitochondrial DNA

## Abstract

Skeletal muscle aging and sarcopenia result in similar changes in the levels of aging markers. However, few studies have examined cancer sarcopenia from the perspective of aging. Therefore, this study investigated aging in cancer sarcopenia and explored its causes in vitro and in vivo. In mouse aging, in vitro cachexia, and mouse cachexia models, skeletal muscles showed similar changes in aging markers including oxidative stress, fibrosis, reduced muscle differentiation potential, and telomere shortening. Furthermore, examination of mitochondrial DNA from skeletal muscle revealed a 5 kb deletion in the major arc; truncation of complexes I, IV, and V in the electron transport chain; and reduced oxidative phosphorylation (OXPHOS). The mouse cachexia model demonstrated high levels of high-mobility group box-1 (HMGB1) and tumor necrosis factor-α (TNFα) in cancer ascites. Continuous administration of neutralizing antibodies against HMGB1 and TNFα in this model reduced oxidative stress and abrogated mitochondrial DNA deletion. These results suggest that in cancer sarcopenia, mitochondrial oxidative stress caused by inflammatory cytokines leads to mitochondrial DNA damage, which in turn leads to decreased OXPHOS and the promotion of aging.

## 1. Introduction

Cancer cachexia is a multifactorial syndrome characterized by persistent skeletal muscle wasting and weight loss [1]. It can also be considered a metabolic syndrome, as it causes disorders and imbalances in glucose, amino acids, fatty acids, bile acids, ke-tone bodies, steroids, and mitochondrial energy metabolism [2,3,4].

Cancer cachexia occurs in 80% of patients with advanced cancer and is associated with 40% of cancer deaths [5,6,7,8]. Cancer cachexia leads to decreased treatment resistance and poor disease prognosis [9,10,11,12]. Sarcopenia, defined as a loss of muscle mass and function, is the most important phenotype of cancer cachexia. Overcoming cancer sarcopenia has become the focus of cancer treatment in improving treatment response, patient prognosis, and quality of life [1,13,14].

At the root of metabolic disorders in cancer sarcopenia is mitochondrial dysfunction [15], and many fundamental cellular processes in muscle tissue, such as apoptosis, autophagy, reactive oxygen species signaling, and protein balance, maintained by mi-tochondria, are impaired [15]. In particular, oxidative stress and imbalanced redox in mitochondria form a vicious cycle, leading to intensified mitochondrial separation, suppression of mitochondrial fusion/fission, inhibition of the electron transport chain, reduced ATP production, increased mitochondrial DNA damage, and impaired mitochondrial biogenesis, playing an important role in muscle tissue damage [6,16,17]. Sat-ellite cells, which play a stem cell-like role in skeletal muscle metabolism, are impaired in function by inflammation, oxidative stress, and fibrosis [18].

Loss of muscle mass and strength is common in older adults and is associated with increased dependency, frailty, and mortality [19,20]. Thus, aging is an important cause of sarcopenia [21,22], but the underlying molecular mechanisms have similarities with cancer sarcopenia. Skeletal muscle loss occurs due to an imbalance between protein synthesis and degradation, increased apoptosis of muscle cells, and reduced regenerative capacity [22]. In terms of mitochondrial dysfunction, both share a common phenotype, including decreased OXPHOS, increased mitochondrial ROS, and impaired mitochondrial quality control [6,23]. Furthermore, mitochondrial DNA damage has been detected in aging, cancer, and neurodegenerative diseases, and has been noted as a common cause of tissue damage [24]. Although the involvement of inflammatory cytokines plays an important role in cancer sarcopenia [25,26], increased levels of inflammatory cytokines have also been observed in older adults and are correlated with sarcopenia [27].

Considering these findings, it is hypothesized that cancer-associated sarcopenia is a state of accelerated skeletal muscle aging. Therefore, in this study, we aimed to clari-fy the involvement of aging in cancer-associated sarcopenia.

## 2. Results

### 2.1. Age-Related Changes of Mouse Skeletal Muscle

First, the effectiveness of various aging markers was examined in the quadriceps femoris muscle (QCM) of mice aged 4 (young), 55 (middle-aged), and 110 weeks (old) (Table 1). The markers included oxidative stress (malondialdehyde [MDA], 4-hydroxynonenal [4HNE], and advanced glycation end products [AGE]), an inflammatory cytokine (high-mobility group box-1 [HMGB1]), aging-related proteins (collagen III and β-galactosidase [βGAL]), telomere volume as a marker for telomere shortening, and skeletal muscle maturation (sodium dodecyl sulfate-soluble myosin light chain-1 [SDS-MYL1]). The levels of all markers increased with age, with increased changes from 55 to 110 weeks than those from 4 to 55 weeks.

### 2.2. Changes in Aging Marker Levels in an In Vitro Cachexia Model

We previously established an in vitro cancer cachexia model (CX) in which cancer ascites were added to the culture medium and demonstrated that the data correlated well with animal cancer cachexia models [28]. Using the CT26 in vitro cancer cachexia model with C2C12 mouse skeletal myoblasts, we examined the changes in the levels of aging markers in a cancerous environment (Table 2). As with aging mice, we examined the same aging markers in the mouse aging model. After 2 days of culture, the levels did not differ significantly between the control and CX groups; however, after 6 days of culture, we observed changes in all markers in the CX group. In the aging mouse model, the levels of oxidative stress, inflammatory cytokines, and aging-related proteins increased, whereas telomere volume and skeletal muscle maturity decreased. These changes in the CX model corresponded to those observed in the senescent stages of mouse aging models, suggesting the promotion of skeletal muscle aging in cancerous environments.

### 2.3. Muscle Cell Kinetics in an In Vitro Cachexia Model

**Figure 1:**
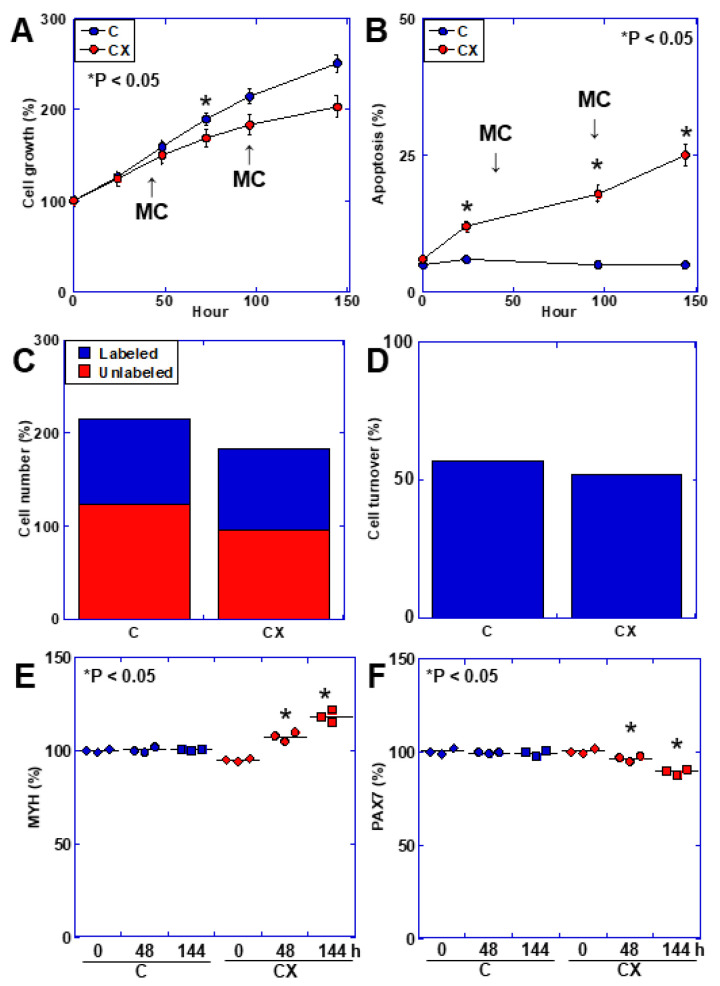
Alterations of cell kinetics and maturation in the in vitro cachexia model. C2C12 cells were treated with a medium containing cancerous ascites (20% *v*/*v*). The medium was changed to ascites-supplemented medium every two days. (**A**) Cell growth. (**B**) Apoptotic cells. (**C**) Number of cells showing cell surface labeling at 144 h. (**D**) Cell turnover (%) calculated as the unlabeled cell number/total cell number. (**E**,**F**) Protein levels of MYH8 and PAX7. * Error bars: standard deviation from three independent trials. Statistical differences were calculated using analysis of variance (ANOVA) with Bonferroni correction. C, control; CX, in vitro cachexia model; MC, medium change; MYH8, myosin heavy chain-8; PAX7, paired box protein-7.

Next, we examined changes in the kinetics of C2C12 cells using an in vitro CX model (Figure 1). The CX model showed decreased proliferative activity (Figure 1A) and increased apoptosis (Figure 1B). The cell turnover was slightly lower in the CX model than that in the control group (Figure 1C). The levels of myosin heavy chain-8 (MYH8), which indicates skeletal muscle regeneration ability, and paired box protein-7 (PAX7), which indicates differentiation ability, were decreased in the CX model (Figure 1D,E).

### 2.4. Mitochondrial Alterations in C2C12 Cells in an In Vitro Cachexia Model

**Figure 2:**
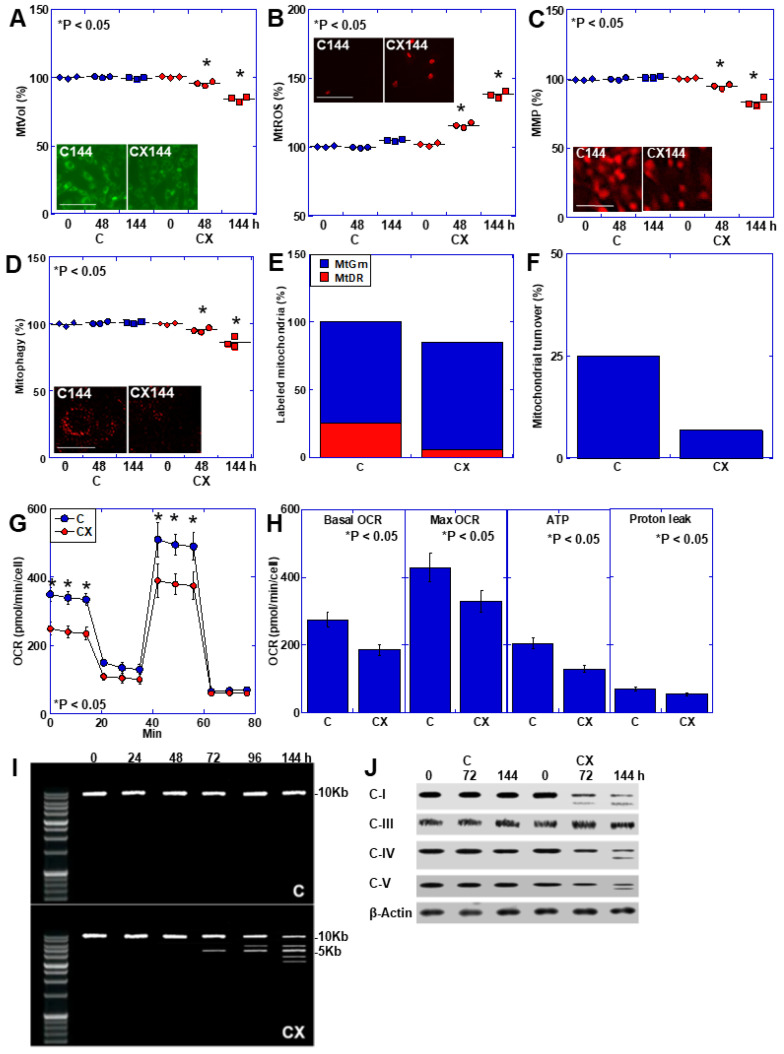
Mitochondrial alterations in the in vitro cachexia model. C2C12 cells were treated with medium containing cancerous ascites (20% *v*/*v*). The medium was changed to ascites-supplemented medium every two days. (**A**) MtVol. (**B**) MtROS. (**C**) MMP. (**D**) Mitophagy. (**A**–**D**) Insert, fluorescence images of C 144 h and CX 144 h. Scale bar 50 μm. (**E**) Labeled mitochondria at 144 h. Mitochondria were labeled by MtGrn at 0 h and relabeled by MtDR at 144 h. (**F**) Mitochondrial turnover (%) calculated by MtDR+/(MtGrn+ + MtDR+). (**G**) Mitochondrial respiration. (**H**) OXPHOS parameters. (**I**) Mitochondrial DNA alterations. Mitochondrial major arc DNA was amplified using PCR. 10 kb, which is considered a normal-sized band. (**J**) Protein levels of the ETC complexes. * Error bars: standard deviation from three independent trials. Statistical differences were calculated using analysis of variance (ANOVA) with Bonferroni correction. C, control; CX, in vitro cachexia model; MtVol, mitochondrial volume; MtROS, mitochondrial reactive oxygen species; MMP, mitochondrial membrane potential; MtGrn, mitogreen; MtDR, mito deep red; OCR, oxygen consumption ratio; OXPHOS, oxidative phosphorylation; PCR, polymerase chain reaction; ETC, electron transport chain; C-I, complex I; C-III, complex III; C-IV, complex IV; C-V, complex V.

Mitochondria play an important role in maintaining skeletal muscle [29]. Examination of the mitochondrial changes in the in vitro CX model (Figure 2) revealed decreased mitochondrial volume (MtVol) and mitochondrial membrane potential (MMP) and mitophagy and increased mitochondrial oxidative stress (MtROS) (Figure 2A–D). The CX model showed a 72% decrease in mitochondrial turnover, (Figure 2E), as well as reduced mitophagy (Figure 2F) and mitochondrial respiration, similar to other OXPHOS parameters (Figure 2G,H). Additionally, as deletions occur in mitochondrial DNA with age [30], we investigated the presence of DNA deletions using primers for the mitochondrial major arc DNA, which contains the most deletions [31] (Figure 2I). The control group showed a normal 10-kb product length regardless of the time course, whereas the normal signal became fainter and the deletion signals were approximately 5 kb and increased after 72 h in the CX model. Other faint deletion signals were also observed. Examination of the protein expression levels of electron transport chain (ETC) complexes (C) I, III, IV, and V showed that the normal signal decreased after 72 h in C-I and after 144 h in C-IV and C-V, with the appearance of small signals indicating truncation (Figure 2J). In contrast, we observed no abnormal bands in the control group. Additionally, we observed no abnormalities in C-III, the gene for which is located in the minor arc, in either the control or CX models. Thus, mitochondrial DNA abnormalities were induced over time in the in vitro cachexia model, leading to mitochondrial dysfunction.

### 2.5. Skeletal Muscle Aging Phenotypes in a Mouse Model of Cachexia

We examined the relationship between sarcopenia and aging in a mouse model of cachexia (Table 3). Using 4-week-old mice (control group) as a reference, we compared a parallel control without tumor inoculation (NT) group with a cachexia (CX) group with tumors inoculated intraperitoneally. Both groups were observed for 30 days. Body and QCM weights were lower in the CX group than in the NT group. Additionally, peritoneal tumors and ascites were observed in the CX group. Examination of the same aging markers (Table 1 and Table 2) in the QCM showed no significant differences between the control and NT groups, whereas the CX group showed increased oxidative stress (MDA, 4HNE, AGE), increased HMGB1, increased collagen III and βGAL, decreased telomere volume, and decreased muscle maturation (SDS-MYL1) (Table 3).

### 2.6. Skeletal Muscle Kinetics in the Mouse Cachexia Model

Examination of the kinetics of QCM skeletal muscle cells in the mouse cachexia model revealed decreased proliferative activity and increased apoptosis in the CX group. Additionally, the expression levels of MYH8, which indicates regenerative potential, and PAX7, which indicates differentiation potential, decreased. In contrast, we observed no significant differences between the NT and control groups (Table 4).

### 2.7. Skeletal Muscle Mitochondrial Alterations in the Mouse Cachexia Model

**Figure 3:**
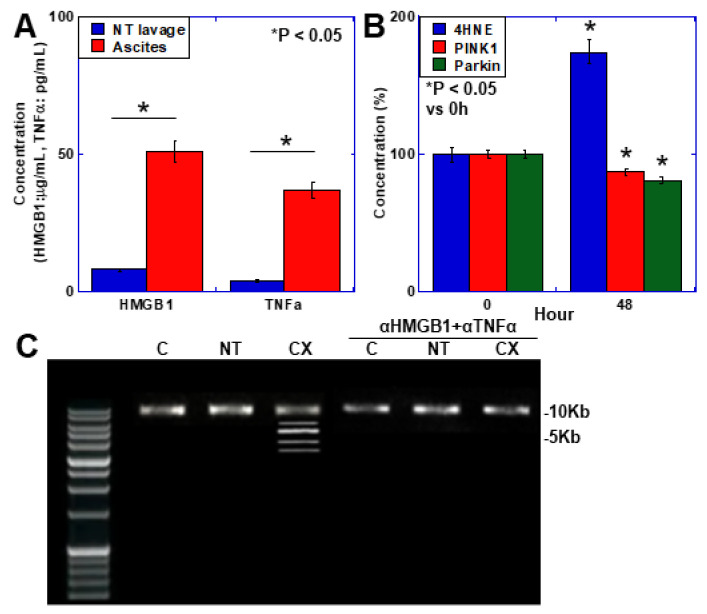
Mitochondrial changes in the skeletal muscle of a mouse cachexia model. (**A**) Concentrations of HMGB1 and TNFα in ascites from CX mice used for peritoneal lavage in NT mice. (**B**) Levels of 4HNE, and protein levels of PINK1 and Parkin in C2C12 cells treated with HMGB1 (50 μg/mL). (**C**) Mitochondrial DNA alterations in the mouse cachexia model. Mice in the αHMGB1 + αTNFα group were injected with αHMGB1 (0.5 μg/mouse) and αTNFα (0.5 μg/mouse) intraperitoneally three times weekly. Mitochondrial major arc DNA was amplified by PCR. 10 kb, normal-sized band. * Error bars: standard deviation from three independent trials or five mice. Statistical differences were calculated using analysis of variance with the Bonferroni correction. C, control; NT, no tumor; CX, cachexia model; 4HNE, 4-hydroxynonenal; PINK1, PTEN-induced putative kinase 1; αHMGB1, anti-HMGB1 antibody; αTNFα, anti-mouse TNFα antibody; PCR, polymerase chain reaction.

Examination of changes in the mitochondria of the skeletal muscle in the mouse cachexia model revealed no significant changes in mitochondrial volume (leucine zipper/EF-hand-containing transmembrane protein-1 [LetM1]), mitophagy markers (PTEN-induced putative kinase 1 [PINK1], Parkin), or OXPHOS markers (phosphocreatine) in the C and NT groups but decreased levels in the CX group (Table 5). Evaluation of the mitochondrial DNA showed normal signals in the C and NT groups and small signals indicating deletions in the CX group (Figure 3). Furthermore, the amount of ETC complex I was decreased only in the CX group (Table 4). Thus, we observed accelerated aging of skeletal muscle in the mouse cachexia model, suggesting mitochondrial dysfunction.

### 2.8. Mechanism of Mitochondrial DNA Damage

As our data suggested that mitochondrial DNA damage affects skeletal muscle aging during cancer cachexia, we used an in vitro cachexia model to investigate the mechanism by which mitochondrial DNA damage was induced (Figure 3).

Measurement of the levels of inflammatory cytokines (HMGB1, tumor necrosis factor-alpha [TNFα]) and lipid peroxides (4HNE) in the cancer ascites (Figure 3A) showed increased levels for all cytokines compared with the control mouse peritoneal lavage. To examine the effect of inflammatory cytokines, we treated the cells with HMGB1 and TNFα for 48 h at concentrations equivalent to those in the cancer ascites (50 μg/mL and 35 pg/mL, respectively) and examined the changes in 4HNE, PINK1, and Parkin (Figure 3B). Both HMGB1 and TNFα treatment results in increased 4HNE levels and decreased PINK1 and Parkin levels. Further examination of mitochondrial DNA deletion in skeletal muscle when the mouse cachexia model was treated with neutralizing antibodies against HMGB1 and TNFα (Figure 3C) showed mitochondrial DNA deletion in the non-antibody-treated group but not in the antibody-treated group.

These findings suggest that increased mitochondrial ROS and decreased mitochondrial quality control owing to inflammatory cytokines induce mitochondrial DNA damage, which leads to skeletal muscle aging through a decrease in mitochondrial function.

## 3. Discussion

In this study, we investigated the relationship between cancer-related sarcopenia and skeletal muscle aging. We observed changes in various aging markers in skeletal muscles in both in vitro and mouse cachexia models, suggesting the promotion of cancer-related aging.

We evaluated markers related to aging in skeletal muscle tissue. A decline in differentiation potential is associated with decreased *PAX7* expression [32] and increased *MYH8* expression [33]. Aging increases collagen I, III, and VI levels in both slow and fast muscles [34]. As indications of mitochondrial changes, decreased mitochondria levels in muscle cells; decreased ETC complexes I, III, IV, and V; decreased mitochondrial transcription factor A (TFAM) levels; OXPHOS inhibition; increased oxidative stress, decreased autophagy, and increased apoptosis have been reported [35,36,37,38]. Telomeres shorten with age and chronic stress promotes these changes [39,40]. AGE are produced non-enzymatically from glucose under oxidative stress but are difficult to degrade; therefore, levels increase with aging and chronic diseases such as diabetes [41]. Although whether such changes in skeletal muscle phenotypes observed in aging were promoted in tumor-bearing models was previously unclear, the levels of all aging markers examined increased in skeletal muscle in in vitro and in vivo cancer cachexia models in the present study. Although determining whether many of these markers are age-specific is difficult, βGAL is the most widely used biomarker for aging and senescent cells [42] and may be a cause of aging [43]. These aging phenotypes may be promoted in cancer cachexia. Comparison of the aged and cachectic mouse models suggested that 8 weeks of age in the cachectic model was equivalent to 110 weeks of age in the aged model, suggesting that the cancerous environment promoted rapid aging of skeletal muscle.

The results of the present study revealed that cachexia causes DNA deletion and dysfunction of skeletal muscle mitochondria. Recent reports have demonstrated the association of mitochondrial DNA deletion with aging. Mitochondrial DNA deletions can be as large as several kilobases and are associated with human pathologies including cancer, aging, and mitochondrial diseases [44]. Mitochondrial DNA deletions lead to the loss of ETC complexes and a general decline in mitochondrial function, including OXPHOS [44]. High-definition sequencing has revealed that various mitochondrial DNA deletions frequently occur with age [31]. In the present study, we observed extensive deletions in the major arc of mitochondrial DNA in cachectic skeletal muscle, leading to mutations in ETC complexes I, IV, and V and suppression of OXPHOS. Simultaneously, mitochondrial ROS levels increased and mitophagy was suppressed. The fact that doxorubicin, which damages mitochondrial DNA, accelerates aging in mice also suggests a relationship between mitochondrial DNA abnormalities and aging [45,46].

Our results also showed that large-scale deletions of mitochondrial DNA increase with age; however, normal mitochondrial DNA signals were also present, indicating mitochondrial DNA heteroplasmy. Heteroplasmy is the phenomenon of mutations coexisting with normal alleles [47]. In this case, OXPHOS disorders are only observed when the proportion of abnormal mitochondrial DNA exceeds a certain threshold [48]. The relationships between mitochondrial DNA heteroplasmy and aging, cancer, and neurodegenerative diseases have attracted considerable attention [49,50]. In the present study, mitochondrial DNA deletions occurred within a relatively short period in the cachexia model, which may be related to a decrease in mitochondrial quality control, leading to the establishment of abnormal mitochondrial DNA. Although we were unable to examine this, mitochondrial DNA point mutations, base modifications, and copy number changes are also mitochondrial DNA changes associated with aging. Future studies examining these changes in cachexia are required.

We showed that increased mitochondrial oxidative stress caused by inflammatory cytokines is involved in the formation of mitochondrial DNA deletions. The causes of large-scale mitochondrial DNA deletions remain largely unknown. Abnormalities in mitochondrial DNA replication, repair, and crosstalk between these pathways are thought to be involved in the generation of large-scale deletions [44]. Mitochondrial DNA is vulnerable to oxidative stress, which induces mitochondrial DNA alterations [50]. Our data also suggest that the generation of mitochondrial ROS by inflammatory cytokines is involved in the development of mitochondrial DNA deletions.

Mitochondrial DNA deletions increase exponentially with age and maps to a wider region of the mitochondrial genome than previously reported [31]. From 50 to 86 years of age, the mitochondrial DNA deletion frequency increases from 0.008% to 0.15% [30]. Furthermore, in skeletal muscle, increasing mitochondrial DNA deletion frequency is correlated with age-related muscle fiber loss and accelerated mortality [51]. Mitochondrial dysfunction induces systemic inflammation by excreting mitochondrial DNA into the cytoplasm or extracellular space and by promoting the secretion of inflammatory cytokines through the nucleotide-binding domain, leucine-rich-containing family pyrin domain-containing-3 (NLRP3) inflammasome in non-immuneand immune cells [52,53,54,55]. In contrast, our data suggest that inflammatory cytokines may increase mitochondrial ROS levels and induce mitochondrial DNA damage. Recent reports have shown that the inhibition of the inflammatory cytokine pathway reduces mitochondrial dysfunction [56]. Thus, mitochondrial dysfunction and inflammatory cytokines may lead to a vicious cycle that exacerbates systemic inflammation and mitochondrial damage. Furthermore, the results of the present study suggest that this cycle promotes tissue aging. In the future, controlling damage-related molecular patterns, including those of inflammatory cytokines and mitochondrial DNA, may help suppress tissue aging.

Our data showed that both in vitro cachexia models and HMGB1 treatment reduced autophagy and the expression of mitophagy-related proteins such as PINK1 and Parkin. Autophagy is an important mechanism for mitochondrial quality control, and its reduction leads to the abnormal accumulation of injured mitochondria in cancer and aging [22,57]. In this study, autophagy was suppressed in cancer cachexia and aging, despite the occurrence of mitochondrial injury that would normally promote mitophagy, such as increased mitochondrial ROS production, decreased MMP, decreased OXPHOS, and mitochondrial DNA deletion. This is thought to perpetuate mitochondrial abnormalities and establish the phenotype of sarcopenia. Furthermore, it has been suggested that mitochondrial dysfunction and inflammation mutually exacerbate each other, leading to the inhibition of autophagy [15]. However, the details of the mechanism have not yet been fully elucidated [15]. Our results show that HMGB1 not only causes mitochondrial dysfunction but also inhibits autophagy and reduces mitochondrial turnover.

In this study, we clarified the commonality of phenotypes between cancer sarcopenia and aging sarcopenia and the underlying mitochondrial disorder. From these results, we considered that cancer sarcopenia is a state in which skeletal muscle aging is accelerated. However, considering that the aging phenotypes are multimodal and cannot be determined solely by limited phenotype, our conclusions are considered to be limited. However, this study was able to shed light on the molecular basis of both aging and cancer, such as the fact that mitochondrial DNA deletion is common to both aging and cancer. In the future, a more extensive comparison of the two conditions will be necessary using various omics analyses. Another limitation of this study is that it was conducted using a culture system and a mouse model, but human analysis was lacking. To prove that the findings of this study can be extrapolated to humans, studies using human samples are essential. By approaching the nature of cancer sarcopenia from the perspective of aging, it is hoped that we can deepen our understanding of not only cancer sarcopenia but also aging itself, and develop treatments for it.

## 4. Materials and Methods

### 4.1. Animals

The aged mouse models were five male BALB/c mice aged 4, 55, and 110 weeks purchased from SLC Japan (Shizuoka, Japan). The animals were maintained in a pathogen-free animal facility under a 12/12 h light/dark cycle in a temperature (22 °C)- and humidity-controlled environment, in accordance with the institutional guidelines approved by the Committee for Animal Experimentation of Nara Medical University, Kashihara, Japan, following current regulations and standards of the Japanese Ministry of Health, Labor and Welfare (approval no. 12777, 20 April 2020). The animals were acclimated to their housing for 7 days before the start of the experiment.

The mouse cachexia model was based on previous reports of the intraperitoneal inoculation of CT26 cancer cells (5 × 10^6^ cells) into syngeneic BALB/c mice (*n* = 5) [28]. Five mice were used as starting controls, and another five were used as non-tumor controls at the time of euthanasia. The mice were euthanized under anesthesia 4 weeks after inoculation. The skeletal muscles were prepared as we previously described [28], and the quadriceps femoris muscle (QFM) was separated from the bones.

### 4.2. Cell Lines and Reagents

The CT26 mouse colon cancer cell line was a gift from Professor I. J. Fidler (MD Anderson Cancer Center, Houston, TX, USA) [58]. C2C12 mouse myoblasts were purchased from Dainihon Pharmacy Co. (Tokyo, Japan). The cells were cultured in Dulbecco’s modified Eagle’s medium (DMEM; Wako Pure Chemical Industries, Ltd., Osaka, Japan) supplemented with 10% fetal bovine serum (Sigma-Aldrich Chemical Co., St. Louis, MO, USA). Human recombinant HMGB1 (Biolegend, San Diego, CA, USA), mouse TNFα (Cell Signaling, Danvers, MA, USA), anti-HMGB1 antibody (clone 3E8, Biolegend), and anti-mouse TNFα antibody (Neutrakine, Proteintech, Rosemont, IL, USA) were purchased from commercial sources.

### 4.3. In Vitro Cachexia Model

For the in vitro cachexia model [59], ascites of CT26 cell-inoculated BALB/c mice were added to supplemented regular medium at 20% *v*/*v*. As a control, regular medium was added to the culture medium of CT26 cells at 20% *v*/*v*.

### 4.4. Protein Extraction

Proteins were extracted from QCM stored at −8 °C as we previously described [28]. Whole-cell lysates were prepared as previously described using radio-immunoprecipitation assay (RIPA) buffer containing 0.1% SDS (Thermo Fisher Scientific, Tokyo, Japan) [60]. Protein assays were performed using a Protein Assay Rapid Kit (Wako Pure Chemical Corporation, Osaka, Japan).

### 4.5. Western Blot Analysis

Protein lysates (25 μg) were separated on 12.5% sodium dodecyl sulfate-polyacrylamide gels, followed by electrotransfer onto a nitrocellulose filter. The membranes were then incubated with primary and peroxidase-conjugated immunoglobulin G (IgG) antibodies (Agilent Technologies, Santa Clara, CA, USA). Immune complexes were detected using an enhanced chemiluminescence (ECL) Western blot detection system (Amersham, Aylesbury, UK). The primary antibodies used in this analysis are provided in Table 1 and were used at a dilution of 1:1000 for immunoblot analysis.

### 4.6. Mitochondrial Imaging

Mitochondrial function was examined using fluorescent probes. After treatment with or without BBR (25 μM), cells were incubated with the probes for 30 min at 37 °C and then photographed using an All-in-One fluorescence microscope (KEYENCE). We used MitoROS (mitochondrial superoxide) (10 μM, AAT Bioquest Inc., Sunnyvale, CA, USA) to assess oxidative stress, mitoGreen (100 nM, PromoCell GmbH, Heidelberg, Germany) to assess mitochondrial volume, and tetramethylrhodamine ethyl ester (TMRE) (200 nM, Sigma-Aldrich) to assess MMP. Mitophagy was detected using a Mitophagy Detection Kit (Dojindo) according to the manufacturer’s instructions.

### 4.7. Enzyme-Linked Immunosorbent Assay (ELISA) and Fluorometric Assay

Whole-cell lysates and mitochondrial fractions were prepared as previously described using RIPA buffer containing 0.1% SDS (Thermo Fisher) [59] and a mitochondrial isolation kit for cultured cells (Thermo Fisher), respectively. ELISA kits were used to measure the protein levels (Table 6). The assay was performed using whole-cell lysates and mitochondrial fractions according to the manufacturer’s instructions.

### 4.8. DNA Isolation

Mitochondrial DNA was isolated from the extracted mitochondria using TRIzol reagent (Invitrogen, Waltham, MA, USA) and purified using an RNeasy Mini Kit (Qiagen, Hilden, Germany) according to the manufacturer’s protocols. Purified DNA was quantified using a NanoDrop ND-1000 spectrophotometer (Thermo Fisher Scientific).

### 4.9. Telomere Quantification

Telomere quantification was performed using a Relative Mouse Telomere Length Quantification qPCR Assay Kit (#M8908, Sciencells Research Lab, Carlsbad, CA, USA) according to the manufacturer’s instructions. DNA sample (2 μg in 1 μL) was mixed with primer stock solution (2 μL), 2 × GoldNStart TaqGreen qPCR master mix (10 μL), and Nuclease-free H_2_O (7 μL). The PCR conditions were as follows: 95℃ for 10 min, followed by 32 cycles of 95 °C for 20 s, 52 °C for 20 s, and 72 °C for 45 s. PCR was performed using an Applied Biosystems QuantStudio Absolute Q digital PCR system (Thermo Fisher Scientific). The comparative ∆∆Cq (quantification cycle value) method was applied to calculate the relative amount of telomeres using the following series of equations:

1. ∆Cq (telomere, TEL) = Cq (TEL, sample 2) − Cq (TEL, sample 1), where TEL = telomere and ∆Cq is the difference in quantification cycles between two samples.
2. ∆Cq (single copy reference, SCR) = Cq (SCR, sample 2) − Cq (SCR, sample 1).
3. ∆∆Cq = ∆Cq (TEL) − ∆Cq (SCR).
4. Relative telomere length of sample 2 to sample 1 (fold) = 2^−∆∆Cq^.

### 4.10. Mitochondrial DNA Mutations

PCR was performed with 0.5 µg DNA extracted from mitochondria. The primer sets used are listed in Table 6 and were synthesized by Sigma Genosys (St. Louis, MO, USA). To amplify long DNA fragments, TAKARA Ex Premier DNA polymerase (TAKARA Bio, Kusatsu, Japan) was used according to the manufacturer’s instructions. The PCR conditions were as follows: 94 °C for 1 min, followed by 30 cycles of 98 °C for 10 s and 68 °C for 5 min. PCR was performed using an Applied Biosystems QuantStudio Absolute Q digital PCR system (Thermo Fisher Scientific). The PCR products were electrophoresed on a 1% agarose gel and stained with ethidium bromide.

### 4.11. Mitochondrial Stress Test (Seahorse Assay)

Mitochondrial and glycolytic stress tests were performed as described previously [60]. The oxygen consumption rate (OCR) and extracellular acidification rate (ECAR) of 1 × 10^4^ viable C2C12 cells per well were measured using a Seahorse XFe24 Extracellular Flux Analyzer with Seahorse XF24 FluxPaks (Agilent Technologies, Chicopee, ON, Canada).

### 4.12. Statistical Analysis

Statistical significances were calculated using unpaired Student’s *t*-tests using InStat, version 3.0 (GraphPad Software, Inc., La Jolla, CA, USA). Data are expressed as the mean ± standard deviation of three independent experiments. Two-sided *p* < 0.05 was considered to indicate statistical significance.

## Figures and Tables

**Table 1:** Age-related changes in aging markers in mouse skeletal muscle.

	4 Weeks	55 Weeks	110 Weeks	*p*
MDA (μg/g)	10.3 ± 0.08	12.2 ± 0.3	14.9 ± 0.3	<0.0001
4HNE (ng/g)	55 ± 0.4	64 ± 0.7	74 ± 1.9	<0.0001
AGE (ng/g)	101 ± 1.1	108.3 ± 2.1	113.0 ± 3.1	<0.0001
HMGB1 (μg/g)	5.2 ± 0.8	7.2 ± 0.8	13.0 ± 1.2	<0.0001
COL (μg/g)	26 ± 0.3	28 ± 0.4	30 ± 0.6	<0.0001
TV (%)	100.0 ± 0.7	95.0 ± 1.2	88.2 ± 1.3	<0.0001
βGAL (ng/g)	7.6 ± 0.08	7.8 ± 0.06	8.6 ± 0.14	<0.0001
SDS-MYL1 (pg/g)	108 ± 0.8	105 ± 0.7	92 ± 0.9	<0.0001
Ki67 (pg/g)	11 ± 0.1	10 ± 0.1	9.3 ± 0.1	<0.0001

Changes in the levels of aging markers in mouse quadriceps femoris (QCM) samples at 4, 55, and 110 weeks of age. Statistical differences (among three groups) were calculated by ordinal analysis of variance (ANOVA) with Bonferroni correction. MDA, malondialdehyde; 4HNE, 4-hydroxynonenal; AGE, advanced glycation end products; HMGB1, high-mobility group box-1; COL, collagen III; TV, telomere volume; βGAL, beta-galactosidase; SDS-MYL1, sodium dodecyl sulfate-soluble myosin light chain-1.

**Table 2:** Changes in the levels of aging markers in in vitro cachexia model in C2C12 cells.

Parameter	0		1 d		6 d	
	C	CX	C	CX	C	CX
MDA (μg/g)	8.1 ± 0.04	8.1 ± 0.1	8.2 ± 0.1	9.5 ± 0.4 *	8.2 ± 0.4	13.2 ± 0.5 *
4HNE (ng/g)	45 ± 0.03	46 ± 0.04	46 ± 0.6	52 ± 1.1 *	47 ± 1.2	64 ± 1.6 *
AGE (ng/g)	82 ± 0.8	81 ± 0.5	83 ± 1.2	87 ± 1.3 *	89 ± 0.9	101 ± 2.0 *
HMGB1 (μg/g)	5.7 ± 0.6	6.0 ± 1.0	7.3 ± 0.6	7.7 ± 0.6	5.7 ± 0.6	15.3 ± 1.5 *
COL (μg/g)	12.5 ± 0.08	12.5 ± 0.08	12.8 ± 0.13	13.2 ± 0.2 *	12.6 ± 0.13	14.6 ± 0.36 *
TV (%)	99.7 ± 0.6	100.0 ± 1.0	98.0 ± 1.0	95.3 ± 1.2 *	97.0 ± 1.0	89.7 ± 0.6 *
βGAL (ng/g)	8.2 ± 0.08	8.1 ± 0.05	8.1 ± 0.05	8.3 ± 0.12 *	8.2 ± 0.08	8.7 ± 0.13 *
SDS-MYL1 (pg/g)	91 ± 0.5	91 ± 00.9	91 ± 0.9	87 ± 1.0 *	92 ± 0.5	78 ± 1.2 *

The levels of aging markers were examined in C2C12 mouse myoblasts cultured in a medium containing cancer ascites fluid (20%, *v*/*v*). * Statistical differences (C vs. CX) were calculated by ordinal analysis of variance (ANOVA) with Bonferroni correction. MDA, malondialdehyde; 4HNE, 4-hydroxynonenal; AGE, advanced glycation end products; HMGB1, high-mobility group box-1; COL, collagen III; TV, telomere volume; βGAL, beta-galactosidase; SDS-MYL1, sodium dodecyl sulfate-soluble myosin light chain-1.

**Table 3:** Changes in the levels of aging markers in skeletal muscle from a mouse cachexia model.

	C	NT	CX	*p*
BW (g)	22.5 ± 0.5	25.3 ± 0.7	18.0 ± 0.5	<0.0001
QCM weight (g)	0.16 ± 0.2	0.17 ± 0.2	0.11 ± 0.1	<0.0001
Tumor weight (g)	−	−	1.4 ± 0.2	−
Ascites (mL)	−	−	4.3 ± 0.5	−
MDA (μg/g)	10.6 ± 0.3	11.1 ± 0.3	23.0 ± 0.8	<0.0001
4HNE (ng/g)	57 ± 0.3	60 ± 2.1	106 ± 4.0	<0.0001
AGE (ng/g)	110 ± 0.6	102.7 ± 3.5	145.3 ± 2.5	<0.0001
HMGB1 (μg/g)	7.0 ± 1.0	7.6 ± 0.6	32.3 ± 2.5	<0.0001
COL (μg/g)	28 ± 0.2	28 ± 0.5	31 ± 0.8	<0.0001
TV (%)	100.0 ± 0.7	99.1 ± 1.5	85.4 ± 2.1	<0.0001
βGAL (ng/g)	7.3 ± 0.08	7.5 ± 0.09	8.9 ± 0.2	<0.0001
SDS-MYL1 (pg/g)	105 ± 0.9	106 ± 1.2	79 ± 1.7	<0.0001

Changes in the levels of aging markers in mouse quadriceps femoris (QCM) in a cachexia model in BALB/c mice, in which syngeneic CT26 mouse colon cancer cells were inoculated into the peritoneal cavity. Statistical differences (among three groups) were calculated by ordinal analysis of variance (ANOVA) with Bonferroni correction. BW, body weight; MDA, malondialdehyde; 4HNE, 4-hydroxynonenal; AGE, advanced glycation end products; HMGB1, high-mobility group box-1; COL, collagen III; TV, telomere volume; βGAL, beta-galactosidase; SDS-MYL1, sodium dodecyl sulfate-soluble myosin light chain-1.

**Table 4:** Changes in skeletal muscle kinetics in a mouse cachexia model.

	C	NT	CX	*p*
Ki67 (pg/g)	10 ± 0.07	10 ± 0.2	8.3 ± 0.3	<0.0001
Apoptosis (per 200 cells)	12.1 ± 1.2	13.4 ± 2.6	32.7 ± 4.3	<0.0001
MYH8 (pg/g)	27 ± 0.2	27 ± 0.3	21 ± 0.5	<0.0001
PAX7 (pg/g)	11 ± 0.07	11 ± 0.2	9 ± 0.2	<0.0001

Changes in the levels of aging markers in mouse quadriceps femoris (QCM) using a cachexia model in BALB/c mice, in which syngeneic CT26 mouse colon cancer cells were inoculated into the peritoneal cavity. Statistical differences (among three groups) were calculated by ordinal analysis of variance (ANOVA) with Bonferroni correction. MYH8, myosin heavy chain-8; PAX7, paired box protein-7.

**Table 5:** Mitochondrial changes in skeletal muscle in a mouse cachexia model.

	C	NT	CX	*p*
LetM1 (pg/g)	28 ± 0.2	28 ± 0.5	21 ± 0.4	<0.0001
PINK1 (pg/g)	6 ± 0.4	47 ± 0.7	39 ± 0.9	<0.0001
Parkin (pg/g)	8 ± 0.9	99 ± 1.5	76 ± 2.0	<0.0001
pCr (pg/g)	31 ± 0.2	31 ± 0.4	20 ± 0.6	<0.0001
C-I (pg/g)	9 ± 0.03	9 ± 0.06	5.8 ± 0.2	<0.0001

Changes in QCM aging markers were examined using a cachexia model in BALB/c mice, in which syngeneic CT26 mouse colon cancer cells were inoculated into the peritoneal cavity. Statistical differences (among three groups) were calculated by ordinal analysis of variance (ANOVA) with Bonferroni correction. LetM1: leucine zipper/EF-hand-containing transmembrane protein 1; PINK1: PTEN-induced putative kinase 1; pCr: phosphocreatine; C-I: complex I.

**Table 6:** PCR primers, antibodies, and ELISA kits.

Target	GenBank		Sequence
Mouse	NC_005089.1	L	CTTCAATCTACTTCTACCGCCGA (5150–5172)
mitochondria		R	AGAGTTTTGGTTCACGGAACA (16245–16265)
Antibody	Clone or Cat#		Company
Mouse C-I	18G12BC2		Abcam, Waltham, MA, USA
Mouse C-III	1F11C4		Proteintech, Rosemont, IL, USA
Mouse C-IV	55082-1-AP		Proteintech, Rosemont, IL, USA
Mouse C-V	A305-417A		Fortis Life Sciences, Waltham, MA, USA
β-actin	ab8227		Abcam, Waltham, MA, USA
Target	Cat#		Company
MDA	ab238537		Abcam, Waltham, MA, USA
4HNE	ab238538		Abcam, Waltham, MA, USA
AGE	LS-F14150		LS Bio, Shirley, MA, USA
HMGB1	LS-F4039		Shino-Test, Sagamihara, Japan
TNFα	#88-7324-88		Thermo Fisher Scientific, Tokyo, Japan
COLIII	abx258006		Abbexa, Cambridge, UK
βGAL	ab119595		Abcam, Waltham, MA, USA
MYL1	orb1211541		Biorbyt, Cambridge, UK
Ki67	EK15089		Signalway Antibody, Greenbelt, MD
MYH8	abx534171		Abbexa, Cambridge, UK
PAX7	MBS2602714		MyBioSource, San Diego, CA, USA
LetM1	abx530900		Abbexa, Cambridge, UK
PINK1	MBS9337825		MyBioSource, San Diego, CA, USA
Parkin	MBS723678		MyBioSource, San Diego, CA, USA
pCr	CB65529263		Chemical Book, Albany. NY, USA
C-I	CSB-EQ027280MO		Cusabio, Houston, TX, USA

C-I, complex I; C-III, complex III; C-IV, complex IV; C-V, complex V; MDA, malondialdehyde; 4HNE, 4-hydroxynonenal; AGE, advanced glycation end products; HMGB1, high-mobility group box-1; TNFα, tumor necrosis factor-α; COLIII, collagen III; βGAL, beta-galactosidase; MYL1, myosin light chain-1; MYH8, myosin heavy chain-8; PAX7, paired box protein-7; LetM1, leucine zipper/EF-hand-containing transmembrane protein 1; PINK1, PTEN-induced putative kinase 1; pCr, phosphocreatine.

## Data Availability

Data are contained within this article.

## Abbreviations

OXPHIOS: oxidative phosphorylation; HMGB1, high-mobility group box-1; TNFα, tumor necrosis factor-α; ROS, reactive oxygen species; QCM, quadriceps femoris muscle; ETC, electron transport chain; TFAM, mitochondrial transcription factor A; NLPR3, nucleotide-binding domain, leucine-rich-containing family pyrin domain-containing-3; cGAS-STING, cyclic guanosine monophosphate (cGMP)-adenosine monophosphate (AMP) synthase (cGAS)-stimulator of interferon genes; C-I, complex I; C-III, complex III; C-IV, complex IV; C-V, complex V; MDA, malondialdehyde; 4HNE, 4-hydroxynonenal; AGE, advanced glycation end products; COLIII, collagen III; βGAL, beta-galactosidase; myosin light chain-1; MYH8, myosin heavy chain-8; PAX7, paired box protein-7; LetM1, leucine zipper/EF-hand-containing transmembrane protein 1; PINK1, PTEN-induced putative kinase 1; pCr, phosphocreatine.
